# Successful Brace Treatment of Pectus Carinatum in Osteogenesis Imperfecta Using the Dynamic Compression System

**DOI:** 10.1055/s-0039-3399557

**Published:** 2019-12-31

**Authors:** Beth A. Orrick, Amy L. Pierce, Charles L. Snyder, Uri S. Alon

**Affiliations:** 1 Department of Pediatric Surgery, Center for Pectus Excavatum and Carinatum, Children's Mercy Hospital, Kansas City, Missouri, United States; 2 Bone and Mineral Disorders Clinic, Division of Pediatric Nephrology, Children's Mercy Hospital, Kansas City, Missouri, United States

**Keywords:** pectus carinatum, osteogenesis imperfecta, dynamic compression system

## Abstract

Osteogenesis imperfecta (OI) is a genetic disorder of collagen resulting in a “fragile” skeleton with increased fracture risk and other complications, dependent on the specific variant. Pectus deformities of the chest wall, while not common, can be associated with OI. The use of a pectus carinatum brace in a patient with OI poses unknown risks for fractures and adverse treatment outcomes. We successfully applied external compression bracing using the dynamic compression system to one such patient. This case illustrates the ability to treat an OI patient with pectus carinatum using a nonsurgical brace, without complications, resulting in an excellent cosmetic result.

## Introduction

Pectus carinatum (PC) is a chest wall deformity resulting in an anterior protrusion of the chest. Correction of PC has historically required a surgical approach, typically with Ravitch repair (resection of deformed costal cartilages, with or without an operative strut). 1 In recent years, external bracing has become the initial treatment of choice for PC in many centers. Since 2011, our center has been successfully correcting PC in children nonoperatively using a brace.

**Fig. 1:**
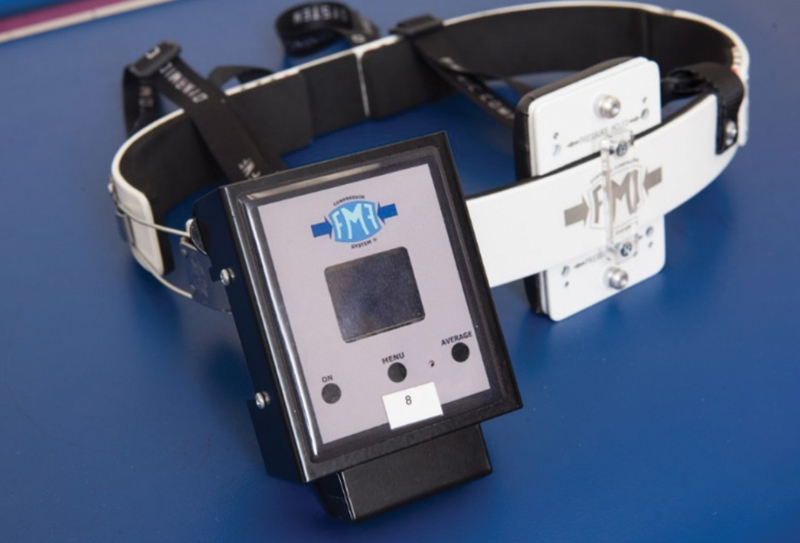
Dynamic compression system (DCS) and pressure measuring device.

Bracing takes advantage of the flexibility of the chest wall in children, allowing reshaping of the sternum and ribs by external compression. A variety of devices and braces are available. We have used the FMF Dynamic Compression System (DCS). 2 (Pampamed Medical Innovations, Buenos Aires, Argentina). The DCS is an external chest compression brace that applies concentrated, measurable pressure to the area of the chest with the greatest protrusion for treatment of PC. At the initial brace fitting, a pressure of initial correction (PIC) is obtained. The PIC is the pressure, measured in pounds per square inch (psi) that is required to completely flatten the carinatum ( Fig. 1 ).

The brace is adjusted to a standard pressure of treatment < 2.5 psi, to minimize skin complications, and maximize brace comfort. 3 The patient is instructed to wear the brace 23 hours per day, removing the brace for sports, bathing, or swimming. PC correction is determined by the patient and provider's subjective assessment of the chest's appearance and neutral sternal position, combined with a PIC of < 1.0 psi. Once correction has been achieved, the brace is worn as a “retainer” to maintain sternal position for shorter durations of time until skeletal maturity has been reached. 2 3 The median duration of therapy until correction is 5.5 months (range: 3.5–10 months). 3

Osteogenesis imperfecta (OI) is a constellation of genetic disorders that primarily affects bone formation. There are at least eight recognized forms of OI, designated type I through type VIII. Type I is the mildest form, and type II is the most severe, with the others ranging between the two extremes. The prevalence is 6 to 7 per 100,000. Types I and IV are the most common forms of OI, accounting for four to five cases per 100,000. 4

Mutations in the COL1A1 and COL1A2 genes (involved in collagen formation) are responsible for > 90% of all cases of OI. 4 Autosomal dominant inheritance is the most common mode of transmission of these genetic abnormalities.

Pulmonary insufficiency and related complications are major causes of morbidity and mortality in OI. The association of OI and chest wall deformities has been reported but is infrequent. 5 6 7 LoMauro et al evaluated pulmonary function in 22 patients with OI and found 7 cases of PC; all had Type III OI. 6

To the best of our knowledge, this is the first case report of successful use of a DCS for the treatment of PC in a child with OI.

## Case Report

After a 5-year hiatus from the Bone and Mineral Disorders Clinic, a 14-year-old male patient with previously diagnosed familial OI type I returned for re-evaluation. At a younger age, he sustained two fragile fractures to his lower extremities and was treated conservatively with casts. Both his father and younger brother are known to have OI. He was never treated with bisphosphonates. Five months before the visit, he sustained a fragile fracture to his right elbow, successfully treated in another hospital. It required open surgery at another institution, with placement of unknown hardware, to be removed 3 months later. At the time of the Bone and Mineral Disorders Clinic visit, he was able to flex and extend the elbow all the way in both directions. His review of systems was unremarkable. His only supplement was vitamin D3 2000 units daily. The family reported their concern of “pigeon chest” appearance that had worsened in the past year. His blood pressure was 115/77 mm Hg, height 164 cm (45th percentile), and weight 46 kg (26th percentile). Physical examination revealed the patient to be well developed for his age with blue sclera, a well-healed surgical scar at the right elbow, and PC.

Laboratory evaluation revealed normal serum concentrations of calcium, phosphorus, creatinine, parathyroid hormone, and 25-hydroxy vitamin D. Bone turnover markers, including bone specific alkaline phosphatase and C-telopeptides and osteocalcin, were high-normal or elevated. The dual-energy X-ray absorptiometry (DXA), which included the spine and distal femurs, revealed Z-scores of -3.0 and lower in all areas studied. Treatment with intermittent zoledronic acid infusions was started the next month. Due to the family's concerns for PC, he was referred to the Pectus Center Clinic.

**Fig. 2:**
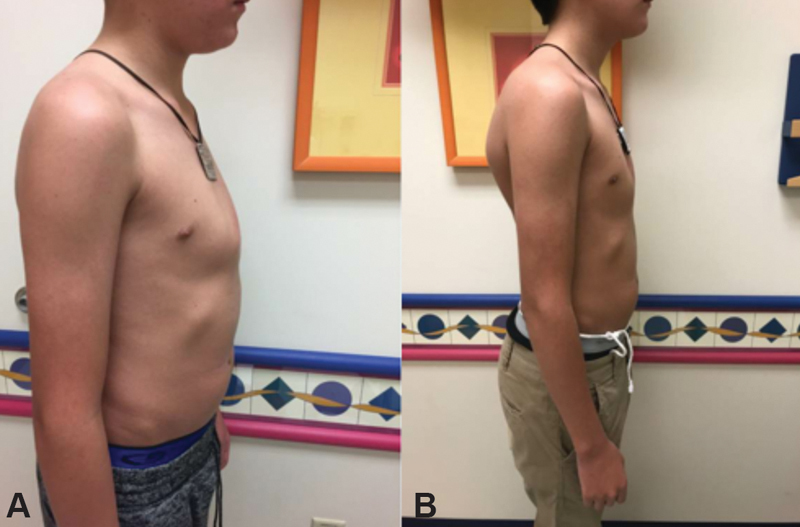
( **A** ) Pectus carinatum before bracing. ( **B** ) Progression of pectus carinatum after 12 months of bracing.

At the Pectus Center, the patient's exam revealed a chondrogladiolar, centrally located, moderate-sized PC that had been present for 1 year with associated chest pain, especially while lying supine ( Fig. 2A ). There were no other known chest wall deformities in the family. A chest X-ray was done per our routine clinical practice, which showed a distal sternal PC deformity with preserved sternal ossification centers. His PIC was 3.7 psi at this initial encounter.

After lengthy discussion of unknown risks as well as the known concern for causing sternal or rib fractures, the patient and his parents were agreeable to begin treatment of PC using a DCS. Further consultation with the bone mineral specialists was performed with a plan to begin three treatment infusions with zoledronic acid (a bisphosphonate derivative) to increase the patient's bone density, followed by attempted carinatum bracing. Once his infusions were complete, the patient was fitted with the DCS with instructions to begin wearing the brace 4 hours per day at a brace pressure of 0.9 psi (lower than usual pressure), then slowly titrate wear time as tolerated to a maximum of 12 hours per day, without sleeping in the brace. If he experienced any pain, he was instructed to remove the brace and notify the pectus clinic. He was initially seen monthly, then every 3 months with an intent to continue visits every 2 to 3 months.

**Fig. 3:**
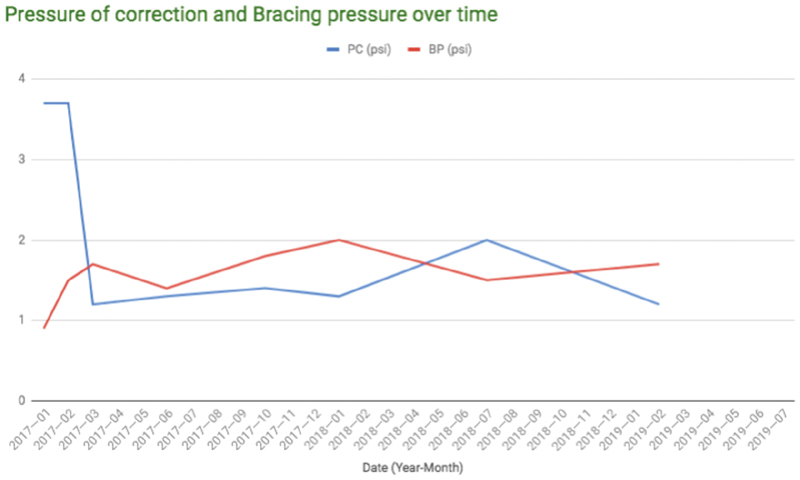
Pressure of correction (PC) and bracing pressure (BP) over time. PC is how much pressure it takes to push the carinatum into a neutral position. BP is how much pressure the brace is applying to the chest during treatment. The BP dropped sharply. The initial PC was lower than that normally used, but was increased slowly since it appeared to be well tolerated.

Follow-up visits in the Pectus Center were not as timely as recommended due to travel difficulties. Nevertheless, the patient remained asymptomatic and reported no chest pain or fractures. His PIC decreased significantly ( Fig. 3 ), with dramatic improvement in the appearance of the PC ( Fig. 2B ). When the patient was next seen in the Bone and Mineral Disorders Clinic for his yearly visit, he still was without new fractures. His blood chemistry was again normal, and his bone turnover markers were all normal. The DXA study revealed a significant improvement, with Z-scores achieving values of –2.2 in the spine, –2.2 in the right femur, and –2.1 in the left femur. Treatment with IV zoledronic acid infusions continued for two more infusions during his brace therapy. At his last Pectus Center visit, we recommended wearing the DCS as much as possible without a time restriction, and at this time, we had braced him for a total of 25 months. We will continue to follow our patient until completion of treatment and to date have found no contraindications to bracing, such as sternal fracture or other adverse events, in a patient with OI.

We define correction of the PC as both a correction pressure of 1.0 psi or less and an acceptable cosmetic result, as determined by the patient and provider. Although our patient has not yet achieved a complete correction, he has made significant progress toward completion with improvement in his PIC from 3.7 to 1.2 psi ( Fig. 3 ). As seen in Fig. 2A , he has achieved an excellent cosmetic result so far. PC patients are typically adjusted to brace pressures of < 2.5 psi. Throughout the brace treatment, we were able to demonstrate that our OI patient was able to tolerate these standard brace pressures. In addition, we conservatively advised to wear the brace fewer hours per day than we would for our non-OI patients.

## Discussion

This case report has demonstrated the ability to successfully treat an OI patient's PC with a DCS resulting in an excellent cosmetic result without complications during the treatment process. To our knowledge, this is the first case report to demonstrate this practice effectively. Although we cannot generalize from a single patient, OI should not be considered an absolute contraindication to correction of PC using a brace. With appropriate bone mineralization management, conservative brace treatment, and close follow-up, successful nonsurgical DCS treatment of a patient with OI and PC is possible. It is important to note that the risk for fracture is unknown. Our patient remained asymptomatic; therefore, radiologic imaging such as chest X-ray or sternal echography was not completed during the bracing treatment. Currently, we are unaware of a way to estimate whether a specific bone density can tolerate a certain amount of pressure. More reports of PC management using a brace in patients with OI are needed to identify success and possible complications such as iatrogenic fractures incurred while bracing.

## Notes

New Insights & the Importance for the Pediatric Surgeon Osteogenesis imperfecta should not be considered an absolute contraindication to nonsurgical correction of pectus carinatum using a brace. With appropriate bone mineralization management, conservative brace treatment, and acceptance of unknown risks for fractures, successful nonsurgical brace treatment of a patient with OI and pectus carinatum is possible.

## References

[1] Martinez-Ferro M Fraire C Bernard S Dynamic compression system for the correction of pectus carinatum Semin Pediatr Surg 2008 17 03 194 200 18582825 10.1053/j.sempedsurg.2008.03.008

[2] Poola A S Pierce A L Orrick B A A single-center experience with dynamic compression bracing for children with pectus carinatum Eur J Pediatr Surg 2018 28 01 12 17 28946165 10.1055/s-0037-1606845

[3] Dekonenko C Dorman R M Pierce A http://doi.org.ezproxy.cmh.edu/10.1089/lap.2019.0171

[4] Osteogenesis imperfecta: National Library of Medicine (US).Genetics Home Reference [Internet]. Bethesda (MD): The Library; 2019 June 11Osteogenesis imperfecta; [reviewed 2013 June; cited 2019 June 12]; [about 6 screens]. Available from:https://ghr.nlm.nih.gov/condition/osteogenesis-imperfecta

[5] Forlino A Marini J C Osteogenesis imperfecta Lancet 2016 387(10028):1657 1671 26542481 10.1016/S0140-6736(15)00728-X PMC7384887

[6] LoMauro A Fraschini P Pochintesta S Romei M D'Angelo M G Aliverti A Ribcage deformity and the altered breathing pattern in children with osteogenesis imperfecta Pediatr Pulmonol 2018 53 07 964 972 29766672 10.1002/ppul.24039

[7] LoMauro A Pochintesta S Romei M Rib cage deformities alter respiratory muscle action and chest wall function in patients with severe osteogenesis imperfecta PLoS One 2012 7 04 e35965 22558284 10.1371/journal.pone.0035965 PMC3338769

